# What are the roles and valued attributes of a Trial Steering Committee? Ethnographic study of eight clinical trials facing challenges

**DOI:** 10.1186/s13063-016-1425-y

**Published:** 2016-07-01

**Authors:** Anne Daykin, Lucy E. Selman, Helen Cramer, Sharon McCann, Gillian W. Shorter, Matthew R. Sydes, Carrol Gamble, Rhiannon Macefield, J. Athene Lane, Alison Shaw

**Affiliations:** MRC ConDuCT Hub for Trials Methodology Research, School of Social and Community Medicine, University of Bristol, Bristol, BS8 2PS UK; Formerly: Health Services Research Unit, University of Aberdeen, Aberdeen, AB25 2ZD UK; Trinity Centre for Practice and Healthcare Innovation, School of Nursing and Midwifery, Trinity College Dublin, Belfast, BT12 6BJ UK; National Institute for Mental Health Research, ANU College of Medicine Biology & Environment, The Australian National University, Canberra, ACT 0200 Australia; MRC Clinical Trials Unit at UCL, London, WC2B 6NH UK; MRC London Hub for Trial Methodology Research, London, UK; MRC North West Hub for Trials Methodology Research, Institute of Translational Medicine, University of Liverpool, Liverpool, L69 3BX UK

**Keywords:** Randomised trials, Good clinical practice, Terms of reference, Trial Steering Committees, Trial monitoring

## Abstract

**Background:**

Clinical trials oversight by a Trial Steering Committee (TSC) is mandated by Good Clinical Practice. This study used qualitative methods to explore the role and valued attributes of the TSC to inform planned updates of Medical Research Council guidance and TSC terms of reference.

**Methods:**

An ethnographic study was conducted during 2013–2014. TSC and Trial Management Group meetings from eight trials were observed and audio-recorded, and semi-structured interviews conducted with purposively sampled key informants: independent and non-independent TSC members, trial sponsor representatives, funder representatives and chief investigators. The selected trials were currently recruiting and dealing with challenging scenarios. Data were analysed thematically and findings triangulated and integrated to give a multi-perspective account of the role and valued attributes of a TSC.

**Results:**

Eight TSC meetings and six Trial Management Group meetings were observed. Sixty-five interviews were conducted with 51 informants. The two main roles played by the TSC were quality assurance and patient advocacy. Quality assurance involved being a ‘critical friend’ or a provider of ‘tough love’. Factors influencing the ability of the TSC to fulfil this role included the TSC Chair, other independent TSC members and the model of the TSC and its fit with the trial subject. The role of the TSC as an advocate for patient well-being was perceived as paramount. Two attributes of TSC members emerged as critical: experience (of running a trial, trial oversight or in a clinical/methodological area) and independence. While independence was valued for giving impartiality, the lack of consensus about its definition and strict requirements of some funders made it difficult to operationalise.

**Conclusions:**

We found tensions and ambiguities in the roles expected of TSCs and the attributes valued of TSC members. In particular, the requirements of independence and experience could conflict, impacting the TSCs’ quality assurance role. Concerns were raised regarding whose interests are served by funders’ criteria of independence; in particular, funders’ selection of TSC members was thought to potentially inhibit TSCs’ ability to fulfil their patient advocacy role. These findings should be incorporated in revising guidance and terms of reference for TSCs.

## Background

Good oversight of late-phase randomised controlled trials (RCTs) is part of the quality assurance process. While there is acknowledged variation in oversight practice within the UK and internationally [1], the UK Medical Research Council (MRC) Guidelines for Good Clinical Practice (1998) recommend that trial oversight should include an element of expert advice that is independent of the Chief Investigator (CI) and host institution involved [2]. This oversight is usually provided by a Trial Steering Committee (TSC).

In the tripartite trial oversight structure recommended by the MRC, the role of the TSC is to act as an executive body providing overall supervision of the trial [2]. The TSC considers the recommendations made by the Data Monitoring Committee (DMC), which reviews safety and efficacy data and steers the Trial Management Group (TMG) responsible for the day-to-day delivery and conduct of the trial. TSCs are expected to monitor and supervise the progress of the trial, review information from other sources (such as related trials), communicate the progress of the trial to relevant parties (such as sponsors and funders) and advise on publicity and presentation of all aspects of the trial [2]. The inclusion in TSCs of members who are independent of both the trial and its TMG is seen as critical in avoiding real or perceived conflict of interest or bias [3] and protecting both trial participants and CIs [4]. The MRC recommends that membership of a TSC should include the CI, who is regarded as being non-independent of the trial, an independent Chair and no fewer than two other independent members [2]. While non-independent members of the trial team can attend TSC meetings, their role is solely to provide information and clarification to independent TSC members.

However, there are apparent inconsistencies or ambiguities in some of the guidance related to TSCs. According to the MRC’s Guidelines for Good Clinical Practice, for example, the TSC prioritises the rights, safety and well-being of the trial participants over the interests of science and society [2], yet its more recent guidance specifies that trial supervision by the TSC is on behalf of the trial Sponsor and trial Funder [4]. Definitions of ‘independence’ as it applies to trial oversight also vary. These include “not involved directly in the trial other than as a member of the TSC” [1], “independent of the investigators, their employing organisations, funders and sponsors” [4] and having no stock ownership in any pharmaceutical company involved, no frequent speaking engagements on behalf of the intervention, no emotional involvement in the running of the trial and not being employed in the same workplace as major members of the trial team [3].

Previous qualitative research in the DAMOCLES project examined the role of DMCs in overseeing trial data collection procedures and advising TSCs [5], resulting in the now widely used 2005 charter for DMCs [6, 7]. However, little attention has been given to the role and function of TSCs and how guidance on TSCs such as that produced by the MRC is applied within trial oversight [7]. Two recent studies have begun to consider these questions [1, 7, 8]. A quantitative survey of 38 UK clinical trials units [7] found that greater clarity is needed regarding the relationship between a TSC and the trial funder or sponsor and the extent of independence required from TSC members. Similarly, the report of Harman et al. of an expert panel review on the role and function of TSCs suggests that the nature of TSCs’ independence, the manner in which TSC members should be appointed and to whom a TSC is responsible are unclear [8]. These study designs, however, did not allow in-depth exploration of the role of TSCs in practice. To complement these studies and inform future revisions of MRC guidance and TSC terms of reference, we aimed to explore the role and valued attributes of TSCs using a multi-perspective ethnographic design.

## Methods

A cross-sectional ethnographic study was conducted, using non-participant observation and interviews to explore the role and value of TSCs. Findings from observation of TSC and TMG meetings were triangulated and integrated with findings from interviews with independent and non-independent TSC members, trial sponsors, funders and CIs to give a rich, multi-perspective account. Our research is situated within a post-positivist, minimally realist paradigm appropriate to applied health services research [9].

### Sample and setting

Based on the research team’s experience and expertise in trials, we hypothesised that the role and value of TSCs would be more clearly articulated in TSCs dealing with challenging scenarios than in other trials. We therefore included trials if they were currently experiencing a difficulty, e.g. recruitment issues, protocol amendments or deviations, participating centres experiencing problems, early release of safety data, early publishing of data or recommendations from the DMC to stop recruitment. A second inclusion criterion was having a TSC meeting planned within the study period (March 2013 – January 2014).

Eight randomised trials meeting the criteria were purposively selected to represent a range of clinical topics (e.g. elderly care, cancer, mental health), healthcare settings (primary and secondary care), interventions (e.g. pharmaceutical and psychological) and oversight committee structures (e.g. dedicated TSC serving one trial or ‘umbrella’ TSC overseeing several trials). The sample size of eight was chosen to enable in-depth fieldwork and analysis across a diverse range of trials. Trials were identified through the UKCRC Registered Clinical Trials Unit Network [10] and a major UK funder of trials. The CTU Network nominated trials and approached trial Chief Investigators (CIs) to invite inclusion of their trial. In addition, the funder identified trials facing difficulties and contacted the CIs with invitations to participate. CIs interested in their trial being included contacted the research team directly.

Purposive sampling was used to select independent and non-independent TSC members with differing perspectives on and involvement in the selected trials. Independent TSC members included TSC Chairs and non-independent members included TMG members. Data collection continued for each trial until data saturation was reached; i.e. no new themes emerged during data analysis [11]. Supplementary interviews were conducted with a purposive selection of trial funder and sponsor representatives and CIs with experience of other challenging trials to gain wider perspectives.

### Data collection

All observational and interview data were collected by the same researcher (AD), a qualitative researcher experienced in health services research.

### Observational data

One TSC meeting was attended, observed and audio-recorded for each trial, with prior consent from TSC members. In addition, to provide a context for discussions within TSC meetings, the researcher attended, observed and audio-recorded relevant TMG meetings during the study period. Detailed field notes were taken during and after the meetings, guided by a standardised observation schedule.

### Interviews

TSC members were invited for interview by the relevant trial CI or by the researcher approaching them directly at meetings. Interviews were conducted before or after the TSC meeting depending on participant availability and preference. Supplementary interviews were conducted with funder, sponsor and CI representatives known to have differing opinions regarding the role and value of TSCs, identified via snowball sampling [12]. All interviews were conducted either face-to-face or by telephone, depending on participant preference, and were recorded and transcribed verbatim.

The semi-structured interview topic guides, formulated on the basis of the literature regarding trial oversight and the research team’s expertise, are summarised in Table 1.

**Table 1:** Interview topic guides

Participant group	Topics discussed in interviews
Chief Investigator	The trial: History of the trial, details of the trial, current stage, successes, current and anticipated challenges
TSC Chair	The TSC: Frequency of meetings, composition of TSC, reasons for selecting members, how Chair was selected and role in meetings, nature of the group’s decision-making and members’ involvement, examples of actioned group recommendations, impact of TSC, communication between TSC and TMG, relationship of and communication between TSC and other trial oversight committees, aspects of TSC that could be improved, recommendations for other trials regarding role of TSC
TSC members	The trial: History of participation in the TSC, views regarding composition of the group and frequency of meetings, relationships with other members, value of TSC meetings, TSC’s role in decisions regarding trial, relationship of and communication between TSC, TMG and other trial oversight committees
	TSC meetings: Meeting organisation, Chair and leadership of meeting, communication during meeting, process of decision-making (positive aspects and challenges/difficulties, own and others’ contributions to decision-making), process of agreeing and assigning actions, communication of actions to other groups/trial personnel, how future TSC meetings could be improved
Trial funder representatives	Funders’ expectations and views of TSCs, process of selecting TSC, examples of TSC working well, examples where TSCs haven’t worked well, different models of TSCs, role of TSC Chair, role of Patient and Public Involvement (PPI), role of the trial funder, regulatory bodies, recommendations regarding TSCs
Sponsor representatives	Sponsors’ expectations and views of TSCs, role of sponsor in trial, responsibilities of sponsor, relationship between sponsor, TSC and funder, challenges faced by trials, qualities of a TSC Chair, decision-making by a TSC, value of TSCs

### Data analysis and rigour

Observational data (meeting recordings and field notes) and interview data were analysed using thematic analysis [13]. A preliminary analysis was conducted by the primary researcher (AD) alongside data collection, to enable data gathered earlier on to inform subsequent data collection. Through a combination of deductive line-by-line coding, based on the research aims, and inductive analysis, an initial coding framework was developed using techniques of constant comparison [14]. Members of the research team (AS, AL, SM, GS and HC) read and independently coded a sub-set of interview and meeting transcripts and met monthly to review data analysis and emerging findings. During the meetings the coding frame was refined into broader categories and higher-level recurring themes, and data within themes were scrutinised for disconfirming and confirming perspectives. Finally, a narrative summary of the findings which integrated data from the interviews and observations was constructed (LS, AD). Triangulation attended to areas of divergence and convergence in the datasets and the different perspectives represented.

The research team represents a range of disciplines and expertise: qualitative health researchers, trial methodologists, statisticians and social scientists with backgrounds in psychology, anthropology and health services research. Several team members had experience of being on TSCs and were known to some of the participants; however, the researcher who collected data had no prior relationships with the participants. One of the informants interviewed was a member of the study team and is a co-author on this paper, but was not involved in data analysis or interpretation. Reflexivity was fostered by the researcher taking detailed field notes on the research process [15] and group discussion of the context of knowledge construction at the monthly team meetings [16]. Data analysis was managed in NVivo v.10, which helped ensure auditability [17].

In presenting findings, data have been anonymised to protect confidentiality. ID codes and trial number are used to identify quotes from interviews. Observational data from meeting recordings and field notes are dated, stating trial number.

## Results

Eight trials participated, all of which were currently recruiting. Six eligible trials were approached via the CTU Network; the CI of one of these refused to participate, as the Chair of the TSC did not want observers at their first ever meeting, in which building relationships was key. The five other trials participated. In addition, of the 32 trial CIs contacted by the funder, five contacted the researchers regarding the study. To fulfil our recruitment target of eight trials, three of these were purposively selected to participate.

We observed and audio-recorded eight TSC meetings and six TMG meetings, totalling 14 hours, 45 minutes. Meetings ranged from 40 minutes to 2 hours in length. We conducted 65 interviews with 51 individuals, who were either members of the eight trials’ TSCs or TMGs or were other relevant informants working in this field (Table 2). No one approached refused to be interviewed. Interviews ranged from 19 minutes to 2 hours, 28 minutes (mean 58 minutes). The median number of interviews per trial was 10, range 6–11. The ratio of independent to non-independent members of TSCs interviewed for each trial ranged from 1:2 to 1:6.

**Table 2:** Characteristics of interview participants

Participant ID	Role	Gender	Trial(s) involved in (subject area), where applicable
01	TSC Chair (Clinician)	M	1, 2 (oncology)
02	Senior trial project lead	M	1, 5 (oncology)
03	TSC coordinator #1	M	1, 2 (oncology)
04	TSC coordinator #2	F	1, 2 (oncology)
05	Sponsor representative	F	1, 2 (oncology)
06	Sponsor representative	M	1, 2 (oncology)
07	Trial manager	F	1 (oncology)
08	CI	F	2 (oncology)
09	Trial manager	F	2 (oncology)
10	Trial manager	M	3 (arthritis)
11	Senior statistician	M	3 (arthritis)
12	Senior trial manager	F	3 (arthritis)
13	Statistician	M	3 (arthritis)
14	CI	M	3 (arthritis)
15	TSC Chair (Clinician)	M	3 (arthritis)
16	Trial manager	F	4 (frailty)
17	TSC Chair (Methodologist)	M	4 (frailty)
18	CI	M	4 (frailty)
19	TMG Chair	F	4 (frailty)
20	TMG member	F	4 (frailty)
21	Trial manager	F	5 (oncology)
22	Statistician	F	5 (oncology)
23	CI	M	5 (oncology)
24	Independent TSC member	M	5 (oncology)
25	Independent TSC member	M	5 (oncology)
26	Trial manager	F	6 (urology)
27	Trial manager	F	6 (urology)
28	Statistician	M	6 (urology)
29	CI	M	6 (urology)
30	TSC Chair (Clinician)	M	6 (urology)
31	Independent TSC member	M	6 (urology)
32	TMG member	M	6 (urology)
33	Trial manager	F	7 (psychology)
34	CI	F	7 (psychology)
35	PPI Representative	M	7 (psychology)
36	TSC Chair (Clinician)	F	7 (psychology)
37	Independent statistician	M	7 (psychology)
38	TSC member	M	7 (psychology)
39	CTU Director	F	7 (psychology)
40	Senior trial manager	F	7 (psychology)
41	Trial manager	F	8 (oncology)
42	Chair (Clinician)	M	8 (oncology)
43	PPI representative	F	8 (oncology)
44	Senior statistician	M	8 (oncology)
45	Sponsor representative	M	8 (oncology)
46	CI of other trial/member of TSCs	M	n/a
47	Funder representative	M	n/a
48	Sponsor representative	M	n/a
49	Funder representative	F	n/a
50	Senior statistician	F	n/a
51	Funder representative	F	n/a

Two main themes with sub-themes emerged: the role of the TSC (quality assurance and patient advocacy) and valued attributes of TSC members (experience and independence).

### Roles of the TSC: scientific quality assurance and patient advocacy

#### Scientific quality assurance

The overall role of the TSC was defined in terms of assuring the rigour and quality of trials; for example, giving “an independent and knowledgeable view” (05, Sponsor representative, trials 1 and 2) of trial progress to guide the trial towards analysis and publication. Participants reported that to fulfil this role, the TSC had to be able to objectively criticise the trial and hold the trial team to account while also providing support and ensuring criticism is constructive. Several interviewees (47, Funder representative; 40, Senior trial manager, trial 7; 39, CTU Director, trial 7) used the phrase ‘critical friend’ to describe this role, while others emphasised the criticising or challenging component of the role; 20, TMG member, trial 4) labelled it ‘tough love’ (Table 3). One TSC Chair (42, trial 8) saw the primary and ethically correct role of an independent TSC as that of a ‘critical advisor’ who would be willing to be a ‘non-friend’ and give unpalatable advice when a trial faces difficulties.

**Table 3:** TSC as ‘critical friend’, provider of ‘tough love’ or ‘critical advisor’: exemplifying quotes

*TSC as ‘critical friend’*	*The ideal function of the Trial Steering Committee [is to] act as a critical friend to the trial, whereby they support the trial to some extent but then they do also ask the awkward questions and hold them to account.* (50, Senior statistician)
	*It was really important to have that external objective view of looking at the data independently, but also, them being our critical friends, advising us and supporting us through this… they’re there to support and help, they’re not just there to chastise.* (40, Senior trial manager, trial 7)
*TSC as provider of ‘tough love’*	*[The role of a TSC] is, I think, tough love. (Laughter)… They’ve got to be on your side… Because if you’ve got a TSC that’s against you, you might just as well hand the money back now. (Laughter)… They’ve got to kind of be in your corner, but I think they've got to be tough.* (20, TMG member, trial 4)
	*If I don’t walk out of these meetings feeling like I’ve been given a bit of a kicking then they haven’t done their job properly, that’s what they’re there to do … it’s their job to… point out the things that we should be doing better.* (23, CI, trial 5)
*TSC as ‘critical advisor’*	Interviewer: *When I’ve asked that question of other people, they value the TSC being a critical friend.*
	42, TSC Chair, trial 8: *No, you can’t. It’s not a friend. A friend implies that the relationship is a good one and always amicable. I wouldn’t hesitate to be a non-friend if I thought it was wrong. Critical adviser – better. However, friend does imply that, “We’ll sit round the table like friends and we’ll just discuss this and what we say will be okay for you.” So criticism; yes, advice; yes. Friendship almost comes as a side issue.*
	Interviewer: *Perhaps the friend bit was them implying that you need to be on their side?*
	42, TSC Chair: *You’re not.*
	Interviewer: *You’re not?*
	42, TSC Chair: *No. You’re independent. So the words of wisdom that you give may be words they don’t want to hear. Maybe we’re going to say, “Right, this trial needs to be shut. It’s not working.” That’s happened three or four times it the last couple of years, in other trials groups. It’s not a matter of cutting your losses. It’s a question of making sure that it’s the ethically proper thing to do. So I’m very keen that meetings are conducted in a friendly environment but we are there as advisers and critics.*

During observations of TSC meetings, the central role of the Chair in enabling the TSC to fulfil its quality assurance role was evident. The TSC Chair of trial 3, for example, seemed to have less control of the proceedings compared with other Chairs, taking the CI’s ”enthusiastic, optimistic” projections of recruitment at face value and skipping items on the agenda to save time, which meant key members of the trial team were not given a voice (field notes, TSC meeting trial 3). In contrast, the Chair of trial 8 (42, quoted in Table 3) dealt with challenges encountered during the trial by “giving timeframes to see improvements” as well as advising about possible solutions (field notes, TSC meeting trial 8).

However, independent members other than the Chair also played an important role in maintaining scientific rigour. The less experienced Chair of trial 7 had a “dream team” with her of two experienced TSC independents who provided “guidance and reassurance” to ensure difficult decisions regarding the trial’s future were made and implemented successfully (field notes, TSC meeting trial 7).

In addition, the model of the TSC and its fit with the trial topic also influenced the ability of the TSC to fulfil its quality assurance role. One of the TSCs in the study was an ‘umbrella’ TSC which oversaw several trials, all in cancer, and reviewed paperwork from eight trials in the 2-hour TSC meeting observed. While the discussion of each trial in this meeting was necessarily brief, the reports submitted by each trial and circulated to the group in advance were noted to be very detailed, with the TSC Chair selecting key elements for discussion. In field notes after the meeting the researcher commented on the efficiency of this model where trials are of a similar nature. Those interviewed from the TSC suggested that the umbrella model would not work well for complex interventions or when the trial itself is complex:

*Where it’s an adaptive trial, lots of different activities, it might be better that we’ve got a TSC that adapts to the trial, similarly if you got a number of different trial groups involved… That’s one reason why you wouldn’t want… to use an umbrella TSC every time. The other is actually is the way that they engage: there is a difference in the way they engage at the two hour meeting, looking just at your trial… compared to a meeting where they meet for 5 or 20 minutes.* (02, Senior trial project lead, trials 1 and 5)

The same respondent highlighted the benefits of the umbrella model:

*Those guys know their stuff, they know their way around the trials pretty well and the issues come up time and time again and they read the papers… There are plusses and minuses of the umbrella approach and the standing trial approach, I’d say. I’ve experience of using both and I think they both work fairly well.* (02, Senior trial project lead, trials 1 and 5)

While the majority of respondents valued the TSC and its contribution to the trial, one CI questioned the scientific worth of the TSC. He acknowledged the value of having a body to provide an ‘external check’ on trial conduct, but in practice thought a TSC had very little impact:

*It seems, to a large extent, to be a rubber-stamping process … an external group checking what you're doing. But I'm not sure it makes a huge difference as to the way the trial runs.* (29, CI, trial 6)

The Chair also felt ambivalent about the value and contribution of his TSC when the trial, he believed, was running smoothly, and hence the TSC had little to contribute:

*I mean, there wasn’t very much on the agenda really, it’s rather formulaic … because it’s going quite well there isn’t a lot to say really.* (30, TSC Chair, trial 6)

In contrast, members of the trial team overseen by this TSC gave specific examples of how the independent TSC members had aided the running of the trial:

*So he (independent member) gave us some great input on improving return rates because he’d worked on a trial where they’d used text messages before.* (28, Senior statistician, trial 6)

The trial manager suggested a reason for this difference of opinion regarding the value of the TSC:

*I think it is because I look at things on a day-to-day level, whereas [CI] perhaps looks at things on a much more broader basis…I value something like an increase in the questionnaire return rate but he (CI) won’t see it as being such a big thing as I do.* (26, Trial manager, trial 6)

These contrasting perspectives highlight the idea that value is judged from a particular viewpoint: different members of the trial team may value different forms of input from a TSC. They also suggest that the role of the TSC may change depending on how well a trial is doing, from rubber-stamping when things are going well to being more critical and directive at other times.

#### Patient advocacy

An additional role of TSCs highlighted in the data was that of patient advocacy. The responsibility of the committee towards patients participating in trials was emphasised in interviews and observed in meetings (“patient value and safety… that’s the guiding thing” (31, TSC Independent member, trial 6)). The role of the TSC as ‘patient advocate’ (42, TSC Chair, trial 8; 45, Sponsor representative, trial 8) was evidenced in phrases such as the patient being the ‘the only reason we’re here’ (01, TSC Chair, trials 1 and 2) and the ‘end point in everything’, ‘the top of the pile’ (42, TSC Chair, trial 8).

During the TSC meeting attended for trial 7, the early stopping of recruitment several months earlier was discussed, and the prioritisation of patient well-being was clear:

37, Independent statistician, trial 7: *That was really the bit I was most interested in… what was going to happen with the patients?*

34, CI, trial 7: *We did have worries. You remember we set up telephone lines and all this.* (Extract from TSC meeting, trial 7)

*What there was complete agreement about, was concern for participants and the welfare of participants in the trial.* (39, CTU Director, trial 7)

The CI described therapeutic alternatives to the trial intervention offered to participants who had not yet started therapy, commenting that “the research therapists were brilliant” (34, CI, trial 7, quoted from TSC meeting). The team discussed a potential future publication on best practice in stopping trials of psychological interventions, given their thorough approach to considering participant well-being.

As this demonstrates, patient advocacy was not distinct from or in conflict with the scientific quality assurance role of the TSC; rather, protecting patient well-being was often perceived as intrinsic to assuring trial quality. However, an independent TSC member highlighted that the roles of scientific quality assurance and patient advocacy might be prioritised differently by TSC members:

*I think in a philosophical way, [the role of the TSC] is to protect the patient, and then the integrity of the trial. I think that should be clear… it puts the interest of the patient first and foremost. As an independent, you are making sure that that is observed.* (37, Independent statistician, trial 7)

The participant differentiated ‘patient safety’ from ‘patient well-being’, arguing that the latter is the remit of the TSC, while the former is the responsibility of the DMC:

*It’s to protect the patient… not just the safety, I think the well-being… because strictly speaking, the safety of the patient is the exclusive reserve of the DMC.* (37, Independent statistician, trial 7)

### Attributes of TSC members

For TSC members, particularly Chairs, to fulfil their roles, two attributes emerged as essential: relevant experience and impartiality through their independence. In particular, both were seen to enhance the rigour of a trial and enable quality assurance. However, while there was broad agreement about the types of experience among TSC members that were of value to a trial, there was less consensus about the meaning of independence and how it could be successfully implemented in practice.

### Experience

Experience in three domains was valued in TSC members: experience of running a trial, experience of sitting on a TSC or DMC and experience or expertise in a particular clinical or methodological area.

Participants acknowledged the complexity of trials and valued TSC members’ prior experience of involvement in or running a trial. The value of experiential knowledge lay in being able to bring practical solutions to problems from their own experience of conducting trials:

*A different view of the world, potentially, but also, ‘Well, when we tried that, it did not work, so we tried this. Maybe you want to give this a try’ … To suggest solutions from your past experience, your past knowledge*. (38, TSC Independent member, trial 7)

Experience of sitting on DMCs or TSCs was also highly regarded by those who were active TSC members. A TSC Chair who was relatively inexperienced appreciated the knowledge and capability of the other two independent members on the committee who were experienced in this role, especially when the trial went through a challenging period. Without their TSC experience, the Chair felt that her role would have been a lot more stressful:

*I probably would have asked permission, probably, to seek advice elsewhere, but [the TSC independent members] were extremely experienced and couldn’t have been better.* (36, TSC Chair, trial 7)

However, as this kind of advice and support are currently provided informally, some interviewees suggested forming a national level advisory board that would advise in complex situations:

*There could be some sort of scientific advisory board, or methodological advisory board… So that when you did hit the buffers, you know… there would be a mechanism for saying, “Look we’re in difficult circumstances here.”… You could actually have an emergency TSC, of the great and the good, to descend on the trial.* (37, Independent statistician, trial 7)

The value of experience in specific clinical and methodological areas was also evident in observational data. In the observed meeting from trial 8, for example, independent TSC members gave advice to the trial team regarding recruitment processes and outcome assessment burden, and informed the less experienced Chair about publication policies.

To ensure relevant experience among TSC members, the Clinical Trials Unit Directors interviewed described informal apprenticeship schemes within their units where those with less trial oversight experience could gain experience of TSCs and access relevant training. There were different models of these apprenticeship schemes, including junior staff taking an observer or non-voting role on a TSC or gaining prior experience on a DMC to improve understanding of committees’ respective roles and responsibilities:

*The easier way to get experience of this, as trial oversight, is as an observer or a non-voting member of a TSC. Then you become a voting member of a TSC. When you’ve got a broad range of experience, you then come on a DMC.* (39, CTU Director, trial 7)

*When you’ve been on a DMC and had really difficult decisions to make, you can kind of appreciate how much is involved and usually how much racking of brains has gone into it. If you’re aware of that from the other side sitting on a TSC you think even harder before you overturn the DMC decision.* (44, Senior statistician, trial 8)

Independent TSC members were in favour of a more formal and national approach to the concept of TSC apprenticeships, which could be incorporated into the continuous professional development of research staff.

*There should be a national bureau or a national register of TSC and DMC members. It should be part of my academic obligation say, to take part in four a year, or something… If we organised it on a national level, and actually had proper training programmes, on the back of that, so that more junior members of staff could get trained up, and kind of get their pilot’s licence — that’s the way to do it.* (37, Independent statistician, trial 7)

Establishing a national apprenticeship system was seen to safeguard the quality of future trial oversight committees and prevent a dearth of suitably experienced people capable of protecting the participants of future trials.

### Independence

#### Valued, but difficult to operationalise

Trial teams valued the independence of the external members of the TSC, associating their distance from the design and day-to-day management of the trial with their ability to give impartial advice. In particular, the absence of a ‘vested interest’ was seen as essential in enabling the TSC to fulfil its patient advocacy role:

*If you have a vested interest in the outcome of the trial as a clinician – trial management, we’ve invested in the protocol, we want to deliver it … I would hope that we would always have the same approach in thinking about patients first, but I think it’s that assurance really, they don’t have invested interest.* (12, Senior trial manager, trial 3)

One CI reflected on how independence provided a valuable check on the emotional and intellectual investment of trial team members, which might put them at risk of prioritising recruitment or early release of data over patient welfare:

*The independence was key, because at that time, I would have done anything to not have the trial stopped. Really, it was correct that it should have been, as per Protocol. Now that things have calmed down, you think, “That is how it should have been.”* (34, CI, trial 7)

The independence of TSC members was also valued by sponsors and funders. Sponsors were conscious of the wider community’s perception of the quality of a trial. They valued independence as it provided a quality kite mark that signalled to outsiders the ‘integrity’ (48, Sponsor representative) of the trial:

*For external purposes it’s important as well, just how it’s perceived by people outside, I think that’s important…for the rigour and for the integrity of the trial*. (48, Sponsor representative)

Funders particularly appreciated independent TSC members during challenging times in the life of a trial, such as when decisions about continuation of the trial were needed. The independent TSC members were considered crucial at such times, given potential biases about trial viability among non-independent members:

*Obviously [for] those that have been involved in developing the study … I think it’s quite difficult to remain unbiased towards continuation of the study.* (49, Funder representative)

However, perspectives varied as to how ‘independence’ should be defined. Funder representatives were aware of the difficulties of implementing independence, particularly in the context of trials within in a specific clinical area, in which the same individuals are involved in multiple roles:

*From a tight knit [clinical] community the same people that are either the chief investigators, serving on a Trial Steering Committee, serving on our Peer Review Committee, are the same people year after year, you know…* (49, Funder representative)

According to some funders’ requirements, independent TSC members should neither be from the same institution as any of the applicants or members of the trial team, nor part of an institution where participants are being recruited. The narrowness of this definition was criticised due to both the difficulty of implementing it and its potential threat to trial conduct. In multi-centre trials involving many known experts in the field, independent members were difficult to find; in the words of one CI, it “really leaves you kind of short of places to look” (29, CI, trial 6). In one trial, an independent member had to resign from the TSC because they changed jobs, disrupting trial oversight:

*We got a new independent member because one of the members … who’s actually been the one who’s made a lot of the good suggestions about what we can do about recruitment and so on… he’s moved here, and somehow or other that’s now a conflict of interest, and I think why? I mean, I still don’t know the bloke and his expertise hasn’t changed just because he’s moved institution, but suddenly he can’t be an independent member.* (29, CI, trial 6)

Some participants argued that institutional affiliation was less of a threat to independence than one’s relationship (e.g. frequent collaborations) with the trial team. While this was acknowledged by a Director within a funding body, he still maintained that employment in a separate institution is the key factor determining independence:

*People are never a hundred per cent independent in the sense that they’re often known to, and in fact they’re nominated by the investigators themselves… but they are nevertheless independent in a sense [that] they’re not from the same institution and certainly aren’t co-applicants and grant holders on the same study.* (47, Funder representative)

Scientific quality of a study was considered at risk when trial teams were required to approach ‘independent’ members outside the specific clinical area and/or who lacked the detailed knowledge that may be helpful to the trial. This could occur when existing experts were judged to be too close to the trial or its team. The requirement of independence thus came into tension with the valued attribute of relevant experience:

*It is difficult to describe the [disease x] clinical community as anything other than a web… everyone’s interconnected in some way… it’s a small world, so if you want that experience, if you want people who know the area, it’s going to be very difficult to get people who aren’t in some way connected.* (02, Senior trial project lead, trials 1 and 5)

*The [funder] fired me [from a DMC], on the basis that by moving back to [city X], I was now at the institution where one of the minor grant holders was… If you insist on that independence, two things are going to happen. First of all, almost by definition, you are going to get someone who is out of the field. However good they are, they’re not going to know about this specific clinical area. Secondly, you run the risk of just getting somebody who isn’t very good.* (38, TSC Independent member, trial 7)

#### ‘Going native’ and the appointment of independent TSC members

The funders interviewed expressed concerns about independent TSC members losing their independence over time through their engagement with the trial and the non-independent members of the TSC. The term ‘going native’ was used to describe their perception of a lessening of impartiality by independent TSC members who became too close to the trial team. This was of disquiet to funders, as they saw the TSC as potentially at risk of colluding with the trial team, reducing the rigour of a trial:

*We use the phrase “TSCs go native”, where they rather forget that they’re there to provide the independent function and…they change into more like the Trial Management Group and see their role as, as there to support the researchers. My own view is that that’s unhelpful.* (47, Funder representative)

Although there were no obvious examples of this in the observational data, as mentioned above, TSC Chairs varied in the extent to which they questioned trial team members, with some accepting their reports at face value and others exhibiting a more critical approach.

For independent TSC members, negotiating their role in contributing expertise to a study, while also remaining independent, could be challenging:

*On the one hand, you don’t want to be completely dogmatic about this… you see a car crash 100 yards down the road, you don’t sit on your hands and say, “Well I’m duty bound not to, you know, not to mention this.” But on the other hand, you do have to be careful, that you don’t roll your sleeves up and without really realising it, change your role, from being an independent trials team committee, to another organ of the research team.* (37, Independent TSC member, statistician, trial 7)

The risk of loss of independence was reported to be a prime motivator for funders seeking control over the appointment of external TSC members and the right to appoint new members. However, TMG and TSC members expressed concerns about this arrangement, questioning whose interests were being served in establishing the TSC, the funder’s or patients’. Independent TSC members and CTU Directors in particular thought that some funders were increasing their control of governance of the TSC while paying inadequate attention to the need for the TSC to be independent from the funder. This was described as shifting the focus away from quality assurance on ‘behalf of the funder’ towards ‘protecting the purse’ of the funder:

*One of the things, I think… that the [funder] needs to grasp, is that it’s not about protecting their purse, because there’s a danger with the way that the [funder] is pushing it: the TSC is seen as protecting the funder rather than holding everybody to account and working to ensure that this is delivered on behalf of the funder. It’s quite a subtle difference… Some TSC chairs, I think, see themselves as closer to the funder than others, and that’s another form of conflict of interest, if you’re too close to the funder. That’s not acknowledged at all.* (39, CTU Director, trial 7)

For some respondents, the nature of the relationship between funder and TSC was therefore critical to the TSC’s ability to fulfil its patient advocacy role. One independent member of a TSC argued that the scientific community, not the funder, should appoint experts to the TSC to uphold the interests of patients:

*The TSC is supposed to be an independent organisation, whereas [a Funding Director] now has very definitely seen it as first and foremost representing the funder’s interest. Now, I said to him, “Look if you just take those letters that you are sending out to all the independents [TSC members] that explains their role, just cross out [funder’s name] and put in [pharmaceutical company’s name], and it will make very uncomfortable reading.”* (38, TSC independent member, trial 7)

## Discussion

This study, the first to explore using qualitative methods the role of TSCs in trials, demonstrates tensions and ambiguities in the roles expected of a TSC and the attributes valued of TSC members. Scientific quality assurance and patient advocacy were identified as the primary roles of the TSC. The quality assurance afforded by the TSC was understood in subtly different ways, reflected in characterisations of the TSC as a supportive ‘critical friend’, a provider of ‘tough love’ or a ‘critical advisor’ not afraid to give unpalatable advice. In general, TSCs were highly valued for the role they played in steering trial conduct, although perceived value could differ between people involved in different capacities in the same trial.

In fulfilling the role of quality assurance, the attributes of experience and independence were seen as critical. Experience enabled TSC members to provide well-informed advice; independence, to remain impartial and balance the potential for biases in the trial team. However, we found that the requirements of experience and independence could conflict. Operationalising funder definitions of independence was difficult, particularly in niche fields of expertise. Here, strict requirements of independence were perceived as potentially detrimental to the running of a trial, preventing the recruitment of TSC members with relevant experience and expertise. Independence, and therefore quality assurance, was also reportedly threatened by TSC members ‘going native’ and losing impartiality. This phenomenon motivated funders’ own selection of independent TSC members. However, the propriety of this involvement by funders was debated, with some seeing independence of the TSC from the funder as critical to the TSC’s ability to fulfil its patient advocacy role.

Findings from this study have clear implications for revisions of the MRC’s guidance and terms of reference for TSCs [1, 2]. The tensions we identified point to a lack of clarity in existing guidance in the following areas:

1. The responsibility of the TSC: Is the TSC’s primary responsibility to uphold the interests of the funder/sponsor, patient, or both? If the latter, then how should the needs of each be balanced? If primary responsibility of the TSC is to the patient, what does this mean for involvement of the funder in trial governance?
2. The meaning of ‘independence’ in the context of a TSC: How should ‘independence’ be operationalised, particularly in narrow specialisms in which relatively few people have relevant expertise and experience?
3. The selection of independent TSC members: Related to item 1 above, what should the role of the funder be in selecting TSC members? Can the interests of patients be upheld if a funder is involved in this choice?

We recommend a revised version of the MRC guidance and terms of reference take into account the current ambiguity and lack of clarity in these areas. Clear guidance, standardisation of processes and transparent decision-making in these areas are needed. The development of consensus about the appropriate definition of independence and strategies for operationalising this when establishing a TSC would contribute towards achieving this. Recent MRC and NIHR guidance clearly indicate a move towards the TSC acting on behalf of the sponsor and funder [4], but debate is needed. Should the TSC first and foremost represent sponsors’ and funders’ interests? Or should its role primarily be as an independent organisation providing overall supervision of the trial? Our study participants gave particular weight to the ‘patient advocate’ role of the TSC, even above their recognised role in ensuring trial integrity [18], and differentiated between the responsibility of the DMC for patient safety and the responsibility of the TSC for patient well-being. However, ensuring patient well-being is not currently identified in the MRC Guidelines as one of the key responsibilities of the TSC [2]. Updates to TSC terms of reference should consider giving greater prominence to the promotion of patient well-being during the oversight process.

This study has strengths and limitations which should be considered in interpreting the transferability of our findings. We utilised multiple ethnographic methods, which allowed an in-depth exploration of the role of TSCs from several perspectives that would not be possible using quantitative or single methods alone. In particular, our observational data demonstrated how differences in chairing styles impacted the ability of a TSC to provide quality assurance and gave insight into how the patient advocacy role of the TSC plays out in a TSC meeting. The eight UK trials in our study are not representative of all randomised trials: seven were funded by one UK funder of trials and the other by a charity; trials funded through other sources and in other countries may face different issues. Furthermore, we selected trials experiencing challenges, and believe this gave us added insight into the role of TSCs. However, an interesting area for future research would be to compare our findings with the reported roles and value of TSCs in trials which are not undergoing such difficulties, as experiences and opinions may differ.

Our findings support and add to the findings from the expert panel of Harman et al. [8]. We found the nature of independence to be a key ambiguity in the characterisation of TSCs. The experts in Harman’s study, like the respondents in this study, raised the question of to whom the TSC was primarily responsible and how this influenced their independence. Ensuring relevant experience and skills among TSC members was also raised in both studies, suggesting that capacity building is an area that requires attention as the field develops. Participants in our study discussed informal apprenticeship schemes designed to ‘train up’ less experienced staff who would become the independent TSC members of the future. To ensure the consistent training of potential independent TSC members, a formal national apprenticeship scheme was suggested. Such a scheme could work if the processes and time required for apprentices to observe and reflect on TSC meetings with appropriate mentors are clearly articulated and factored into the funding of trials. The apprenticeship scheme would provide experiential ‘on-the-job’ training of TSC functioning, and not just classroom-based teaching of particular skills (e.g. statistics).

We found that three types of experience among TSC members were valued: experience of running a trial, experience of sitting on a TSC or DMC and experience or expertise in a particular clinical or methodological area. All three of these areas of experience were stated as requirements of TSC members in a recent survey of registered CTUs [7]. However, this study provides further insight as to why trial teams value these types of experience. Where TSC Chairs or independent members lacked this experience, participants suggested that a national Advisory Board would be useful to advise independent TSC members in complex situations, e.g. when facing challenging decisions such as stopping a trial.

Further research is needed to grow the evidence base regarding trial oversight. In particular, the relationships between TSC members, the TMG and DMC, and the role of the TSC Chair emerged as important in understanding trial oversight in practice; the DAMOCLES project reported similar findings in relation to DMCs [6]. Further in-depth investigation in these areas is warranted. In addition, it was beyond the scope of this paper to explore the role of PPI representatives in trial oversight and conduct, and future research in this area is needed [19].

## Conclusions

This qualitative study of the TSCs of eight clinical trials facing challenges revealed that independent TSC members provide impartial and informed advice to improve scientific rigour and to act as trial participants’ advocates to ensure their well-being. However, we found tensions and ambiguities in the roles expected of TSCs and the attributes valued of TSC members. In particular, the requirements of both independence and experience could conflict, impacting the quality assurance role of the TSC. Although independence of TSC members is valued, operationalising funders’ definitions of independence can generate logistical issues. Concerns were raised regarding whose interests are served by funders’ criteria of independence; in particular, funders’ selection of TSC members was thought to have detrimental ramifications for the TSC’s patient advocacy role. These findings should be incorporated in revising guidance and terms of reference for TSCs.

## Abbreviations

CI, Chief Investigator; DMC, Data Monitoring Committee; EME, Efficacy and Mechanism Evaluation Programme; MRC, Medical Research Council; NHS, National Health Service; NIHR, National Institute for Health Research; TM, Trial Manager; TMG, Trial Management Group; TSC, Trial Steering Committee.
